# ADDRESSING COMORBID CHRONIC PAIN AND EARLY COGNITIVE DECLINE IN OLDER ADULTS: THE ACTIVE BRAINS EFFICACY TRIAL

**DOI:** 10.1093/geroni/igad104.1799

**Published:** 2023-12-21

**Authors:** Ryan Mace, Ana-Maria Vranceanu, Nathaniel Choukas, Elizabeth Rochon, Katherine McDermott, Julia Hooker, Christine Ritchie

**Affiliations:** Massachusetts General Hospital/Harvard Medical School, Boston, Massachusetts, United States; Massachusetts General Hospital, Boston, Massachusetts, United States; Massachusetts General Hospital, Boston, Massachusetts, United States; Massachusetts General Hospital, Boston, Massachusetts, United States; Massachusetts General Hospital/Harvard Medical School, Boston, Massachusetts, United States; Massachusetts General Hospital/Harvard Medical School, Boston, Massachusetts, United States; Massachusetts General Hospital, Boston, Massachusetts, United States

## Abstract

Chronic pain (CP) and early cognitive decline (ECD) are costly, highly prevalent, and challenging to treat among community-dwelling older adults. CP and ECD commonly co-occur, exacerbate each other, and can accelerate dementia. Our interdisciplinary team has developed Active Brains (AB), a virtual group mind-body walking program aided by Fitbit, to address this comorbidity. We are conducting a single-blinded, NIH stage 2 RCT to establish the efficacy of AB versus a time- and dose-matched education control (Health Enhancement Program; HEP) in 260 older adults with CP and ECD. In this symposium, we will describe the study design, recruitment numbers, and maximizing the inclusion of racial and ethnic minoritized older adults (38% benchmark). Participants are recruited through providers, social media, and community partnerships. Participants wear an ActiGraph to monitor steps and are randomized to 8 weeks of AB or HEP delivered via Zoom by an MGH psychologist. The AB group receives a Fitbit to reinforce activity goals. Primary (multimodal physical function) and secondary (cognitive and emotional function, pain) outcomes are collected at baseline, post-intervention, and 6 months. We have recruited 37 participants (4 cohorts). All 37 completed the baseline, 26 completed the post-test, and 9 are currently enrolled (total dropout = 5%). Session attendance (92.8%) and watch adherence (93.5%) have been excellent. The trial will establish efficacy, test mechanisms, and inform a future multi-site effectiveness-implementation trial. Consistent with the GSA 2023 theme, AB has the potential to catalyze non-pharmacological interventions for CP–ECD and empower older adults to maintain active and meaningful lives.

